# Spatiotemporal air pollution exposure assessment for a Canadian population-based lung cancer case-control study

**DOI:** 10.1186/1476-069X-11-22

**Published:** 2012-04-04

**Authors:** Perry Hystad, Paul A Demers, Kenneth C Johnson, Jeff Brook, Aaron van Donkelaar, Lok Lamsal, Randall Martin, Michael Brauer

**Affiliations:** 1 School of Population and Public Health, University of British Columbia, 2206 East Mall, Vancouver, BC V6T 1Z3, Canada; 2 Occupational Cancer Research Centre, Cancer Care Ontario, Ontario, Canada; 3 Science Integration Division, Centre for Chronic Disease Prevention and Control, Public Health Agency of Canada, Ontario, Canada; 4 Air Quality Research Division, Environment, Ontario, Canada; 5 Department of Physics and Atmospheric Science, Dalhousie University, Ontario, Canada; 6 Atmospheric Chemistry and Dynamics Branch, NASA Goddard Space Flight Center, Greenbelt, USA; 7 Department of Physics and Atmospheric Science, Dalhousie University, Canada; Harvard-Smithsonian Center for Astrophysics, Cambridge, USA; 8 School of Population and Public Health, University of British Columbia, Vancouver, BC, Canada

**Keywords:** Air pollution, Canada, Exposure assessment, Lung cancer, Residential mobility, Spatiotemporal

## Abstract

**Background:**

Few epidemiological studies of air pollution have used residential histories to develop long-term retrospective exposure estimates for multiple ambient air pollutants and vehicle and industrial emissions. We present such an exposure assessment for a Canadian population-based lung cancer case-control study of 8353 individuals using self-reported residential histories from 1975 to 1994. We also examine the implications of disregarding and/or improperly accounting for residential mobility in long-term exposure assessments.

**Methods:**

National spatial surfaces of ambient air pollution were compiled from recent satellite-based estimates (for PM_2.5 _and NO_2_) and a chemical transport model (for O_3_). The surfaces were adjusted with historical annual air pollution monitoring data, using either spatiotemporal interpolation or linear regression. Model evaluation was conducted using an independent ten percent subset of monitoring data per year. Proximity to major roads, incorporating a temporal weighting factor based on Canadian mobile-source emission estimates, was used to estimate exposure to vehicle emissions. A comprehensive inventory of geocoded industries was used to estimate proximity to major and minor industrial emissions.

**Results:**

Calibration of the national PM_2.5 _surface using annual spatiotemporal interpolation predicted historical PM_2.5 _measurement data best (R^2 ^= 0.51), while linear regression incorporating the national surfaces, a time-trend and population density best predicted historical concentrations of NO_2 _(R^2 ^= 0.38) and O_3 _(R^2 ^= 0.56). Applying the models to study participants residential histories between 1975 and 1994 resulted in mean PM_2.5_, NO_2 _and O_3 _exposures of 11.3 μg/m^3 ^(SD = 2.6), 17.7 ppb (4.1), and 26.4 ppb (3.4) respectively. On average, individuals lived within 300 m of a highway for 2.9 years (15% of exposure-years) and within 3 km of a major industrial emitter for 6.4 years (32% of exposure-years). Approximately 50% of individuals were classified into a different PM_2.5_, NO_2 _and O_3 _exposure quintile when using study entry postal codes and spatial pollution surfaces, in comparison to exposures derived from residential histories and spatiotemporal air pollution models. Recall bias was also present for self-reported residential histories prior to 1975, with cases recalling older residences more often than controls.

**Conclusions:**

We demonstrate a flexible exposure assessment approach for estimating historical air pollution concentrations over large geographical areas and time-periods. In addition, we highlight the importance of including residential histories in long-term exposure assessments.

For submission to: Environmental Health

## Background

Exposure to ambient air pollution is a suspected risk factor for lung cancer [1-6]. Due to the long latency periods associated with lung cancer, epidemiological analyses are particularly challenging, especially for air pollution where spatial and temporal variation in both residential mobility and air pollution concentrations may produce significant exposure misclassification if not properly incorporated into the exposure assessment approach.

Residential mobility data are required for accurate long-term air pollution exposure assessments, but due to the difficulties in obtaining this information, residential location at study entry or at time of diagnosis is often used to estimate lifetime or long-term exposure estimates in epidemiological studies. Given that approximately half of all individuals move within a five year period [7] and that residential mobility varies depending on socio-economic factors [8-11], there is potential for exposure misclassification and bias in studies that ignore or improperly account for residential mobility. While there is growing recognition of the need for spatiotemporal epidemiology approaches and life-time residential histories in exposure assessment [12], mainly in cancer epidemiology [13,14], little is known regarding the potential exposure misclassification and bias resulting from self-reported residential histories, the most common form of attaining residential histories in epidemiological studies [15], and from the assumption of residential stationarity in air pollution epidemiology.

Incorporating residential histories into air pollution exposure assessments requires corresponding air pollution concentration estimates that cover the spatiotemporal domain of the study period. To date, the association between air pollution and lung cancer has been examined using a variety of study periods and exposure assessment approaches. The most common approaches have aggregated air pollution monitoring levels within cities or defined areas [1,2,6,16], estimated ambient air pollution levels at residential addresses using fixed-site monitoring data or dispersion models [3-5,17,18], or used proximity to roads and industrial sources as exposure surrogates [19,20]. In terms of national retrospective exposure assessment studies, few are available that examine multiple pollutants and exposure sources [21,22].

Here we develop a comprehensive spatiotemporal exposure assessment approach for Canada and apply it to a population-based case-control study of 8353 individuals who provided lifetime self-reported residential histories. For the exposure period 1975 to 1994, we assign fine particulate matter (PM_2.5_), nitrogen dioxide (NO_2_) and ozone (O_3_) air pollution exposures, as well as exposures to vehicle and industrial emissions. The implications of disregarding and/or improperly accounting for residential histories in long-term exposure assessments are also examined. The exposure assessment methods developed produce annual spatiotemporal exposure estimates and will allow subsequent epidemiologic analyses to examine latency periods, to include both urban and rural populations, and to study the contributions of multiple ambient pollutants and local vehicle and industrial emissions to lung cancer risk in Canada.

## Methods

### The lung cancer case-control study

We utilize the lung cancer component of the National Enhanced Cancer Surveillance System (NECSS), which includes 3280 histological-confirmed lung cancer cases and 5073 population controls collected between 1994 and 1997 in the provinces of British Columbia, Alberta, Saskatchewan, Manitoba, Ontario, Prince Edward Island, Nova Scotia and Newfoundland. The respective ethics review boards of each province reviewed and approved the NECSS study. Due to residential mobility, study participants are located in all provinces of Canada requiring national-level exposure assessment. Johnson et al. [23] describe the overall recruitment methodology for the NECSS. Briefly, cases were identified through provincial cancer registries and mailed a research questionnaire. The response rate for contacted lung cancer cases was 61.7%. Population controls were selected from a random sample of individuals within each province, with an age/sex distribution similar to that of all cancer cases (strategies for recruiting population controls varied by province depending on data availability and accessibility). Provincial cancer registries collected information from sampled controls using the same protocol as for the cases. The response rate for contacted population controls was 67.4%.

Residential histories at the 6-digit postal code level are the basis of the air pollution exposure assessment reported here. In urban areas a 6-digit postal code typically incorporates one side of a city block, but represent substantially larger areas in rural locations (e.g. greater than 100 km^2 ^in remote locations of Canada). Residential histories were converted to postal codes by the Public Health Agency of Canada and geocoded using DMTI Inc. 1996 postal codes. While lifetime residential histories were collected, the exposure period was restricted to 1975 to the start of study enrolment (1994), due to the presence of recall bias in earlier reported histories (explained in more detail in the discussion section) as well as the lack of information on postal code locations, air pollution monitoring data and geographic information prior to 1975.

### Air pollution exposure assessment approach

A multi-staged approach was required to assign ambient air pollution concentrations to residential histories from 1975 to 1994. The spatiotemporal exposure assessment included three steps. First, national spatial surfaces were created from recent satellite-based estimates (for PM_2.5 _and NO_2_) and a chemical transport model (for O_3_). Second, all National Air Pollution Surveillance (NAPS) monitoring data were compiled and formatted for the study period, including 120 NO_2 _stations and 1030 measurement-years, 187 O_3 _stations and 1440 measurement-years, 177 TSP stations and 1826 measurement-years, and 25 PM_2.5 _stations and 141 measurement-years. Due to the small number of PM_2.5 _measurements available, and no measurements made prior to 1984, a random effect model was used to estimate PM_2.5 _based on TSP measurements and metropolitan indicator variables. Finally, the spatial pollutant surfaces were calibrated yearly to estimate average annual concentrations between 1975 and 1994. Two approaches were used for calibration: the first estimated historical annual averages using smoothed inverse distance weighting (IDW) interpolation of the ratios of spatial co-located historical NAPS and surface estimates, while the second used linear regression models.

Exposure to vehicle emissions was estimated using proximity to highways and major roads, adjusted based on historical vehicle emissions in Canada. Exposures to industrial emissions were calculated based on proximity to major and minor industrial sources extracted from a comprehensive database of industrial facilities in Canada operating during the study exposure period. Estimates for different vehicle and industrial emission sources were not converted into concentrations and added to ambient concentration estimates as we want to examine each source and distance threshold separately in subsequent epidemiological analyses. Specific components of the exposure assessment approach are described in detail below.

### National spatial pollutant surfaces

**Figure 1:**
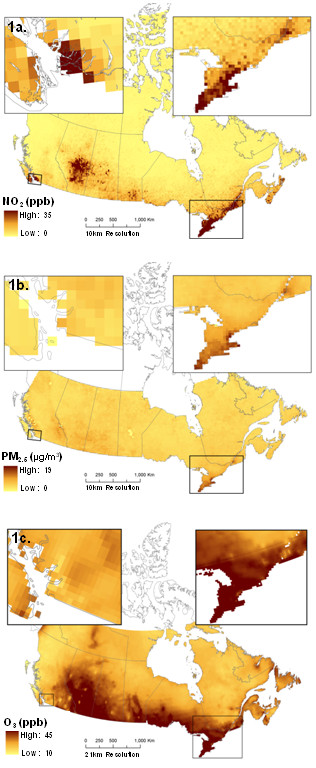
**National pollutant surfaces created from recent satellite estimates (for PM_2.5 _and NO_2_) and a dispersion model (for O_3_)**. Insets represent higher population density locations in Canada (south western BC and southern Ontario and Quebec).

Spatial models of ambient PM_2.5_, NO_2 _and O_3 _concentrations were developed to represent current spatial pollution patterns across Canada. A PM_2.5 _surface was derived from Aerosol Optical Depth (AOD), using data from the Moderate Resolution Imaging Spectroradiometer (MODIS) and the Multiangle Imaging Sectroradiometer (MISR) satellite instruments, and was combined with a chemical transport model (GEOS-Chem; http://www.geos-chem.org) to estimate the relationship between aerosol optical depth and surface PM_2.5 _(for full details see [24]). Estimates for PM_2.5 _represented a composite estimate developed from 2001 to 2006 and included locations with greater than 100 valid measurements to ensure estimate representativeness. The NO_2 _surface was estimated from tropospheric NO_2 _columns retrieved from the Ozone Monitoring Instrument (OMI) and also used GEOS-Chem to calculate the relationship between the NO_2 _column and surface NO_2 _[25]. NO_2 _estimates used data from 2005 to 2007 as OMI measurements began in late 2004. Both PM_2.5 _and NO_2 _were estimated at a 0.1 × 0.1 degree resolution (~10 × 10 km). The O_3 _surface was created from the Canadian Regional and Hemispheric O_3 _and NO_x _System (CHRONOS) [26]. This model is reinitialized every 24 h with meteorology and is fused with the O_3 _observations across Canada and the U.S. on an hourly basis using an optimal interpolation approach based upon a least square combination of the CHRONOS and measured O_3 _data that minimized the error variance. This surface was created at a 21 km resolution and represents average summer (May through September) concentrations from 2004 to 2006. Figure 1 illustrates the PM_2.5_, NO_2 _and O_3 _pollutant surfaces used to represent current spatial concentrations across Canada. Next, these surfaces were calibrated with NAPS monitoring data to estimate historical annual spatial exposure surfaces.

### Air pollution monitoring data

**Figure 2:**
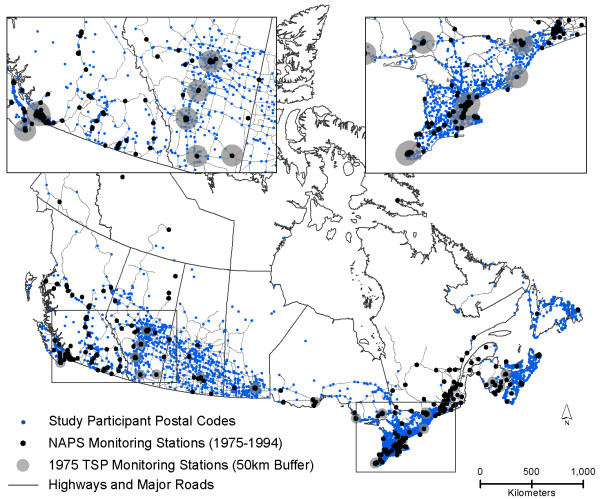
**Location of all national air pollution surveillance monitors in Canada and study participant residential postal codes between 1975 and 1994**.

The NAPS monitoring network began measurements of TSP in 1970, NO_2 _and O_3 _in 1975 and PM_2.5 _and PM_10 _in 1984. Figure 2 illustrates the location of all NAPS monitors in Canada, 1975 TSP monitoring stations with 50 km buffers (for reference of historical monitor spatial coverage) and all study participant residential postal codes between 1975 and 1994.

NAPS monitoring data were first formatted into monthly averages for all pollutants. Continuous monitoring data were included if at least 50% of daily hourly observations were available and at least 50% of days were available in a month. Monthly averages from dichotomous samplers (PM_2.5_) required a minimum of 3 of 5 valid monthly measurements. Yearly averages were not calculated unless there were at least six months of complete data with one month per season, and summer O_3 _averages unless there were 3 months of data available. Supplemental material, Figure1 illustrates historical annual average pollutant concentrations from available NAPS monitoring stations that were in operation for all years. Temporal trends show a large decrease in TSP concentration during the study period (51% from 1970 to 1994), a decrease in NO_2 _(28% from 1975 to 1994) and PM_2.5 _(32% from 1984 to 1994), and an increase in O_3 _(19% from 1975 to 1994). Importantly, the changes in pollutant concentrations were not uniform across geographic areas in Canada.

#### Modeling historical PM_2.5 _concentrations from TSP

**Figure 3:**
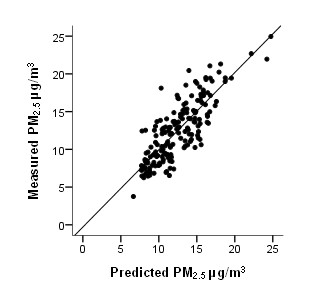
**Correspondence between predicted PM_2.5 _concentrations using TSP concentrations and metropolitan indicator variables and NAPS PM_2.5 _measurements**.

Due to the lack of historical spatial and temporal PM_2.5 _measurement coverage, we used co-located PM_2.5 _and TSP measurements between 1984 and 2000 to create predictive models of historical PM_2.5 _concentrations. The overall approach to estimating PM_2.5 _is similar to that used by Lall et al. [27] to estimate metropolitan area specific PM_2.5 _and PM_10 _relationships with TSP across the U.S. We used random effect models (GLIMMIX procedure in SAS 9.3) to account for the clustering of annual measurements over time at each NAPS station. Table 1 summarizes the final PM_2.5 _model incorporating TSP concentrations (μg/m^3^) and census metropolitan area (CMA) indicator variables. The R^2 ^and RMSE for the PM_2.5 _model was 0.67 and 2.31. Figure 3 illustrates the measured and predicted PM_2.5 _concentrations. The resulting PM_2.5 _model was applied to all valid TSP monitoring stations; the nearest CMA core within 100 km was used to determine the CMA model coefficient for the PM_2.5 _model, otherwise no CMA variable was included in the model. Figure 2 in the supplemental material maps the CMA's used in the model and areas covered by the 100 km buffers.

**Table 1:** Model used to predict historical PM_2.5 _using TSP measurements and census metropolitan area indicator variables (R^2 ^= 0.67, RMSE = 2.31).


**Variables**	**Estimate**	**SE**	**p**

**Intercept**	1.93	2.30	0.42

**TSP**	0.13	1.78e^-2^	< 0.001*

**CMA Indicator**			

Calgary	0.44	2.63	0.87

Edmonton	-1.82	2.69	0.50

Halifax	7.71	3.02	0.01*

Hamilton	4.76	3.02	0.12

Montreal	6.01	2.42	0.01*

Ottawa	4.86	2.94	0.10

Quebec	3.17	2.60	0.23

St. Johns	5.72	3.81	0.13

Saint John	3.28	30.7	0.29

Toronto	5.63	2.60	0.03*

Vancouver	6.50	2.47	0.01*

Victoria	2.48	2.73	0.36

Windsor	5.63	2.56	0.03*

Winnipeg	1.00	-	-

**Model performance: **R^2 ^= 0.67, RMSE = 2.31.R^2 ^ and RMSE estimated by regressing the predictions from the fixed-effects terms against measured values

*Significant at p < 0.05

### Calibrating spatial pollutant surfaces using historical data

Two approaches were used to extrapolate current PM_2.5_, NO_2 _and O_3 _surfaces to estimate annual concentrations between 1975 and 1994. Both approaches were developed using 90% of the monitoring data available for each year, while retaining 10% for model evaluation. Model performance was assessed using adjusted R^2 ^and root-mean-square error (RMSE).

The first approach calibrates the current spatial surfaces (shown previously in Figure 1) using annual NAPS monitoring data and smoothed IDW interpolation of the ratio's of spatial co-located historical NAPS and surface estimates. The yearly calibrations were performed using the following equation:

(1) $$Yearly\;Historical\;Surface_{j}=Surfac{e_{x,y}}^{\times }\left[\frac{\Sigma_{k=1}^{N_{naps}}\left(\frac{1}{\left(d_{x,y,k}\right)}x\frac{\bar{\text{NAP}{\text{S}}_{k}^{J}}}{Surface_{k}}\right)}{\Sigma_{k=1}^{{}_{naps}}\frac{1}{\left(d_{x,y,k}\right)}}\right]$$

Where for each year between 1975 and 1994 the annual historical surface for pollutant *j *is equal to the current spatial surface of pollutant *j *(*Surface_x,y_) *at coordinates *x,y *multiplied by the IDW interpolation of the ratio's of spatial co-located historical NAPS and surface estimates. *d_x,y,k _*is the distance (km) from NAPS monitoring station *k *to location *x,y*. $\bar{NAPS_{K}^{J}}$ and *Surface_k _*are coincidently sampled pollutant concentrations of *j *at station *k*. A smooth interpolation option (smooth factor = 0.2) was included in the IDW interpolation (not shown in equation 1 for simplicity), which uses three ellipses in the interpolation method: points that fall outside the smaller ellipse but inside the largest ellipse are weighted using a sigmoid function [28]. The smoothed IDW function was used to reduce abrupt changes in the yearly calibration surfaces as these do not reflect spatial patterns of pollution change.

The second approach uses linear regression to model annual concentrations. Predictor variables include the spatial pollutant surfaces, a time-trend and historical population density data. Population location data were derived from the 1971, 1976, 1981, 1986, 1991, and 1996 Canadian census; between census years were assigned the nearest census. The annual population density variables were calculated in a GIS for various buffer distances (1 km to 50 km's) around each NAPS monitor. Roads and industry were not included in the models as we want to separately evaluate exposure to these sources and lung cancer risk. We used random effect models (GLIMMIX procedure in SAS 9.3) to account for the clustering of annual measurements over time at each NAPS station and selected predictor variables that maximized model fit. We estimated R^2 ^and RMSE statistics by predicting the measurement data with the fixed-effect coefficients using ordinary least squares regression.

### Exposure to vehicle emissions

Exposures to vehicle emissions were estimated using proximity measures to highways (freeways and major highways) and major roads (freeways, highways, and arterial and collector roads). The 1996 DMTI Inc. road network was used to derive proximity measures for all case and control residential years, due to the lack of historical national road networks. The average distance to each road class was calculated separately as well as the number of years residing within 50, 100 and 300 m of a highway and/or major road. These proximity distances were selected as vehicle related pollutant gradients, such as for NO_2 _and volatile organic compounds, are highest within 50 and 100 m of a major road but remain significantly elevated to 300 m [29].

Emissions from vehicles have changed significantly over time due to increases in vehicle kilometres travelled and improved vehicle emission controls [30,31]. Exposure indicators for years residing near highways and major roads were therefore weighted to account for these changes. Supplemental material, Figure 3 shows the decrease in the total NO_x _emissions from on-road mobile sources in Canada (used here to represent primary vehicle emissions), including heavy and light duty diesel and gasoline vehicles, from 1980 to 2007 and extrapolated levels to 1970. NO_x _emissions estimates were compiled by Environment Canada using the latest emission estimation methodologies and statistics available as of March 2008. Emission factors were developed using MOBILE6.2 C and the number of vehicle kilometres travelled. MOBILE6.2 C is a vehicle emissions modeling software specific to Canada and accounts for the vehicle fleet profile, vehicle emission standards, and fuel characteristics [32]. Given the NO_x _emissions trends documented in the United States from 1970 to 1980 [33], linear extrapolation was used to estimate NO_x _emissions from 1980 to 1970. The ratio of resulting 1994 and 1975 NO_x _emission estimates suggest that living near a major road in 1975 is equivalent to 1.26 "1994" years due to changes in vehicle emissions (the ratio also accounts for changes in vehicle numbers). A weighting factor (1 + 0.013*(1994-proximity exposure year)) was therefore used to adjust proximity-based vehicle exposures to account for decreases in the magnitude of vehicle emissions over the study period.

### Exposure to industrial emissions

A comprehensive inventory of industrial emissions sources was compiled as part of the NECSS within the Environmental Quality Database (EQDB) [23,34,35]. Locations of industrial manufacturing facilities and activities in approximately fifty standard industrial classifications (SIC) from 1970 to 1994 are included in the database along with operational time periods. Approximately 7800 sources with a 4 digit SIC are included and 8200 municipal waste facilities. Major industries, including metal smelters, pulp and paper mills, petroleum product companies, foundry and steel plants, aluminum smelters, non-hydro power plants, and petrochemical companies, contain pollutant discharge estimates while minor industrial sources have no emission records. The distance between an industrial source and a subjects' postal code has been validated to +/-150 m in urban locations [34]. The EQDB has been used in conjunction with the NECSS to examine leukemia and chlorination by-products [36] and residential proximity to industrial plants and Non-Hodgkin's Lymphoma [37]. We calculate exposure to major industrial emissions and to minor sources within 1, 2 and 3 km buffers from residential postal codes. These distances were selected to ensure specificity of proximity based exposure assessments for multiple industries and substances. Similar distance thresholds have been used previously in small area health studies [38,39]. To be considered exposed, and to calculate the number of years exposed to each proximity category, at least 1 industrial facility had to be operating within the associated buffer distance.

## Results

### Residential histories

The NECSS questionnaire asked participants to list each place in Canada that they had lived for at least one year. A total of 8176 individuals (98%) reported at least one full 6-digit postal code and 6918 individuals (83%) reported at least 15 years of residential histories from 1975 to 1994. On average, individuals reported 2.3 (SD = 1.6) different residences from 1975 to 1994; 1617 individuals lived only in rural areas and 4222 individuals lived only in urban areas of Canada. Urban areas were defined using Statistics Canada community size classifications (urban core, urban fringe, urban areas outside of CMA, rural fringe, and rural areas outside of CMA). In total, 77% of the studies exposure-years occurred in urban areas.

**Figure 4:**
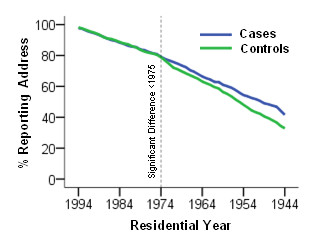
**Percent of cases and controls reporting residential addresses at the 6-digit postal code level from the start of study enrollment (1994) to1944**.

Importantly, while no significant difference (p = 0.54) was found in the number of geocoded residential-years between cases and controls for the 1975 to 1994 exposure period, cases tended to report older addresses more often than controls. Recall bias was especially evident for residential histories prior to 1975, as shown in Figure 4.

### Ambient exposure assessments

**Figure 5:**
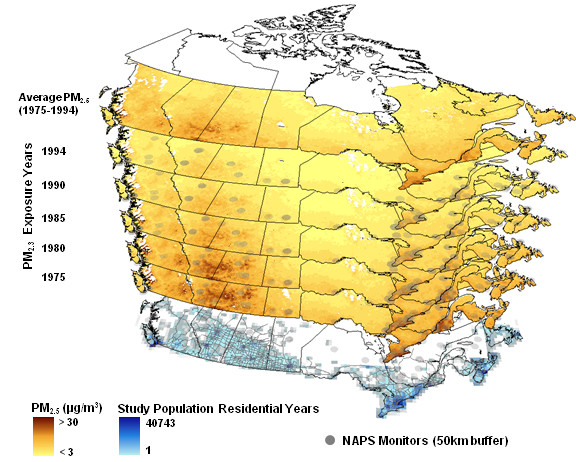
**Example of annual PM_2.5 _exposure surfaces created using the IDW interpolation calibration approach for all years between 1975 and 1994**.

The first approach to calibrating current pollution surfaces used IDW interpolation to create annual surfaces between 1975 and 1994. Figure 5 illustrates the resulting PM_2.5 _exposure surfaces for 1975, 1980, 1985, 1990 and 1994, PM_2.5 _measurement locations with 50 km buffers, the average PM_2.5 _exposure surface between 1975 and 1994, and the location of the case-control study subjects. Twenty annual exposure surfaces were created from 1975 to 1994, but only five are shown here. The study population residential years indicates the locations of all yearly residential histories during the twenty year exposure period summed within a 50 km grid. The temporally adjusted surfaces for NO_2 _and O_3 _are provided in Figures 4 and 5 of the supplemental material.

The performance of the linear regression models was moderate for all three pollutants (PM_2.5 _R^2 ^= 0.33, NO_2 _R^2 ^= 0.36 and O_3 _R^2 ^= 0.47) as described in Table 2. Population density within 10 km of monitoring stations was most strongly associated with PM_2.5_, while population density with 5 km was most strongly associated with NO_2 _(positively associated) and O_3 _(negatively associated). A linear time-trend did not improve the O_3 _model and was therefore not included in the final model.

**Table 2:** Results of historical PM_2.5_, NO_2 _and O_3 _linear regression models.

Model	Distance	Value	SE	p
**PM_2.5 _Model [R^2 ^= 0.33, RMSE = 3.57]**				

Intercept	-	1.18	1.16	0.31

Satellite PM_2.5_	-	0.46	0.11	< 0.001

Population Density	10 km	3.94e^-6^	2.89e^-7^	< 0.001

Years < 1994	-	0.29	9.28e^-3^	< 0.001

**NO_2 _Model [R^2 ^= 0.36, RMSE = 7.00]**				

Intercept	-	10.88	1.07	< 0.001

Satellite NO_2_	-	1.67	0.46	< 0.001

Population Density	5 km	2.6e^-5^	5.11e^-6^	< 0.001

Years < 1994	-	0.28	0.028	< 0.001

**O_3 _Model [R^2 ^= 0.47, RMSE = 5.13]**				

Intercept	-	6.85	1.66	< 0.001

O_3 _Dispersion Model	-	0.73	0.06	< 0.001

Population Density	5 km	-2.0e^-5^	2.5e^-6^	< 0.001

Evaluation of the two historical calibration approaches are shown in Table 3 which summarizes the R^2 ^and RMSE of model evaluations using the 10% sample of monitoring data withheld each year. The spatiotemporal IDW interpolation of PM_2.5 _had the best performance (R^2 ^= 0.51), while the NO_2 _and O_3 _linear models had the best performance (R^2 ^= 0.38 and R^2 ^= 0.56). Model performance tended to decrease for older measurements, but not substantially. Additional file 1: Supplemental material 1, Figure 6 presents the scatter plots for each model evaluation.

**Table 3:** Evaluation of spatiotemporal IDW interpolation and linear regression models to predict annual historical air pollution.

				IDW Interpolation		Linear Models	
	**Year**	**Stations**	**N**	**R^2^**	**RMSE**	**R^2^**	**RMSE**

**NO_2_**	All	120	1030	0.22	6.66	0.38	5.92

	1994-1990	94	349	0.30	5.66	0.36	5.42

	1989-1985	88	300	0.20	6.61	0.44	5.54

	1984-1980	62	226	0.13	6.72	0.40	5.62

	1979-1975	52	155	0.17	8.75	0.29	8.07

**PM_2.5_**	All	177	1826	0.51	2.96	0.30	3.53

	1994-1990	106	446	0.64	1.96	0.32	2.70

	1989-1985	113	480	0.57	2.30	0.36	2.81

	1984-1980	124	476	0.34	3.79	0.12	4.36

	1979-1975	123	424	0.43	3.32	0.26	3.77

**O_3_**	All	187	1440	0.39	5.29	0.56	4.48

	1994-1990	158	582	0.53	4.92	0.65	4.25

	1989-1985	125	409	0.36	5.41	0.54	4.57

	1984-1980	80	286	0.25	4.67	0.28	4.57

	1979-1975	48	163	0.22	6.33	0.60	4.50

Table 4 presents the exposure assessment results using both historical calibration methods and air pollution exposures derived from NAPS monitoring data within 50 km of residential postal codes. To ensure accurate exposure assessment, results are presented for individuals with at least 15 complete exposure-years between 1975 and 1994. Exposures for different time-periods (e.g. 1975-1980, 1975-1985, and 1975-1990) were also calculated to examine different latency periods (data not shown).

**Table 4:** Ambient exposure estimates derived from NAPS monitors within 50 km of residential postal codes and spatiotemporal exposure models.

Pollutant	N*	Mean	SD	Min	IQR	Max
**NAPS Measurements ≤ 50 km**						

TSP (μg/m^3^)	4027	60.0	16.9	22.3	21.4	114.1

Modeled PM_2.5 _(μg/m^3^)^a^	4027	17.0	2.5	11.9	3.4	25.7

NO_2 _(ppb)	3649	23.4	6.0	6.0	7.6	37.8

O_3 _(ppb)^b^	4382	21.0	3.9	7.0	5.3	32.6

**Spatiotemporal IDW Interpolation**						

PM_2.5 _(μg/m^3^)	6833	11.3	2.6	3.6	3.9	19.0

NO_2 _(ppb)	6919	15.3	8.8	1.1	14.5	43.4

O_3_^b^(ppb)	6919	23.2	3.7	12.9	4.6	35.4

**Linear Regression Models**						

PM_2.5 _(μg/m^3^)	6833	9.1	1.9	4.7	2.2	16.1

NO_2 _(ppb)	6919	17.7	4.1	13.1	5.0	35.1

O_3_^b ^(ppb)	6919	26.4	3.4	18.1	4.7	37.2

*Number of individuals with ≥ 15 complete exposure-years

^a ^Modeled using TSP and CMA indicator variables as described previously in Table 1

^b ^Summer (May through September) O_3_

### Exposure to vehicle and industrial emissions

Proximity measures used to represent exposure to vehicle emissions are summarized in Table 5. Individuals lived within 50, 100 and 300 m of a highway for a mean of 0.5 (SD = 2.9), 1.1 (SD = 4.0) and 2.9 (SD = 6.3) years, respectively. Exposure years increased slightly when weighted by temporal emission changes. The average mean distance from study participants' postal codes to the nearest highway was 3.9 km. When residential histories were restricted to urban areas (where proximity is a more accurate measure of exposure than in rural areas), the distance to highways and major roads decreased substantially. Over half of the study population was exposed to emissions from a major road at some point during the study period (i.e. had lived at least one year within 300 m of a major road).

**Table 5:** Proximity measures to highways and major roads.

Proximity Measure	**# of People Exposed**^a^	# of Years Exposed (Mean ± SD)	**# of Weighted**^b ^**Years Exposed (Mean ± SD)**
**Highways**			

≤ 50 m	341	0.5 (2.9)	0.7 (3.9)

≤ 100 m	647	1.1 (4.0)	1.5 (5.4)

≤ 300 m	1640	2.9 (6.3)	4.0 (8.5)

**Major Roads**			

≤ 50 m	1438	2.3 (5.5)	3.2 (7.6)

≤ 100 m	2283	4.0 (6.9)	5.5 (9.5)

≤ 300 m	4517	10.1 (8.8)	13.8 (12.1)

^a ^Number of individuals living > 1 year within 50/100/300 m of a highway or major road

^b ^Weighted to account for temporal changes in vehicle emissions

The number of years study participants lived within 1, 2 and 3 km of a major and minor industry are summarized in Table 6 as are aggregated emission estimates for major industrial sources. Proximity to specific emission sources (e.g. oil refineries, smelters, and pulp and paper mills) were also calculated (data not shown). Individuals lived within 1, 2 and 3 km of a major industrial source for a mean of 1.6 (SD = 5.3), 4.3 (8.3) and 6.4 (9.5) years respectively. Over half of the study population (n = 5942) lived within 3 km of a minor industrial source for at least one year between 1975 and 1994.

**Table 6:** Proximity measures to major and minor industrial sources.

Proximity Measure	**# of People Exposed**^a^	# of Years Exposed (Mean ± SD)	# of Facilities (Mean &#177 SD)	**Emissions**^b ^**(tonnes) (Mean ± SD)**
**Major Industries**				

≤ 1 km	838	1.6 (5.3)	6.2 (5.5)	4.5e^5 ^(3.6e^7^)

≤ 2 km	1995	4.3 (8.2)	13.3 (11.6)	4.5e^5 ^(3.5e^7^)

≤ 3 km	2743	6.4 (9.5)	21.3 (18.6)	1.9e^3 ^(1.6e^4^)

**Minor Industries**				

≤ 1 km	4137	11.4 (11.2)	32.6 (59.3)	-

≤ 2 km	5515	16.7 (10.0)	115.7 (163.2)	-

≤ 3 km	5942	18.9 (9.0)	218.0 (303.8)	-

^a ^Number of individuals living > 1 year within 1/2/3 km of a major or minor industrial source

^b ^Summary of facility emissions > 0 tonnes. Only available for major industries

### Disregarding residential histories and exposure error

A total of 3305 study participants (40%) lived at their study entry address for the entire twenty year exposure period, while 622 (7.6%) participants lived for 15-19 years, 970 (11.9%) for 10-14 years, 1433 (17.5%) for 5-9 years, and 1756 (23%) for less than 5 years. Correlation between ambient air pollution exposures derived from study entry residential addresses only, in place of exposures derived from residential histories and spatiotemporal air pollution models, were relatively high for PM_2.5 _r = 0.70, NO_2 _r = 0.76 and O_3 _r = 0.72. However, when examining exposure misclassification based on incorrectly assigned exposure quintiles, 50%, 49% and 46% of individuals where classified into a different PM_2.5_, NO_2 _and O_3 _quintile. When temporal variation is removed from the exposure assessment (i.e. historical exposures are derived from residential histories applied to the current spatial pollution surfaces) 17%, 15% and 14% of individuals where classified into a different PM_2.5_, NO_2 _and O_3 _exposure quintile. Similar results were found for proximity based exposures, for example, 30% of individuals classified as not exposed to highway emissions based on their address at study entry were actually exposed when residential histories were used for exposure assessment.

## Discussion

Incorporating residential mobility in chronic air pollution studies is fundamental to accurate exposure estimates. Boscoe [15] presents a review of environmental health studies that have incorporated residential histories to-date. In our study, only 40% of participants lived at their study entry residence for the entire 20 year exposure period; on average, 2.3 (SD = 1.6) different residences per subject were reported. Recall bias was present for self-reported residential histories prior to 1975, with cases recalling older residences more often than controls. This has important implications for environmental epidemiology using self-reported residential histories as many environmental exposures have decreased substantially over time. Consequently, exposure assessment based on a greater proportion of older residential histories in cases compared to controls will result in an upward bias, rather than non-differential bias typically assumed from exposure misclassification. Studies that incorporate self-reported residential histories, particularity long-term residential histories - in this case over twenty years, may need to account for reporting bias in epidemiological analysis.

This study also demonstrated the importance of estimating air pollution exposures from residential histories, both in terms of including different residential locations as well as the corresponding spatiotemporal air pollution concentration estimates. Exposure quintiles based on residential addresses at study entry had approximately 50% correspondence to exposure quintiles developed from residential histories and spatiotemporal air pollution surface. These results address one of the research opportunities suggested by Meliker and Sloan [12]: "indentifying circumstances under which it is worthwhile to compile and incorporate extensive space-time data histories of mobility or environmental contaminants". Epidemiological studies of diseases with long latency periods (in this case lung cancer) and/or that examine spatially and temporally varying exposures (in this case ambient air pollution) are clearly such circumstances.

Despite the fact that the Canadian NAPS monitoring network is one of the longest-standing national air pollution monitoring programs worldwide and now covers the majority of urban centers in Canada, its limited spatiotemporal coverage necessitated the creation of national models that capture both urban and rural populations. We were able to use NAPS data within 50 km of residential postal codes to assign exposures to 63%, 70% and 54% of exposure-years for TSP, O_3 _and NO_2_. Very limited spatial and temporal PM_2.5 _monitoring data were available (only 40% of exposure-years between 1984 and 1994 could be assigned) and we therefore estimated historical PM_2.5 _using TSP and metropolitan area indicator variables. The resulting models predicted PM_2.5 _variability well; the ratio for modelled PM_2.5_/TSP (0.32, SD = 0.12) is very similar to that found in US metropolitan areas (PM_2.5_/TSP = 0.30, SD = 0.11) [27].

National spatial pollutant surfaces were compiled and calibrated with historical NAPS data to assign ambient pollutant concentrations to all study participants' residential postal codes between 1975 and 1994. The two approaches used to calibrate spatial pollutant surfaces differ in their approach to account for temporal and spatial change; IDW interpolation accounted for the heterogeneity in pollution level changes across Canada during the exposure period, while linear regression models incorporated a linear time-trend and population density as a spatial predictor. The interpolation approach better represented historical PM_2.5 _concentrations, potentially due to the larger spatial scale of PM_2.5_, while the linear regression models better represented historical NO_2 _and O_3 _concentration, which have finer spatial resolutions.

The creation of national spatiotemporal models allowed for the inclusion of all study participants, regardless of geographic location and NAPS monitor coverage. This was important as 42884 (23%) of exposure-years occurred in rural areas. The mean PM_2.5_, NO_2 _and O_3 _exposure estimates derived from the spatiotemporal models were 11.3 μg/m^3 ^(SD = 2.6), 17.7 ppb (4.1), and 26.4 ppb (3.4) respectively. The magnitude of these exposures are less than those used in other studies, for example, the widely cited ACS study (PM_2.5_: 17.7 μg/m^3 ^(3.0), NO_2 _21.4 ppb (7.1); and O_3 _45.5 ppb (7.3)) [1]. This is likely due to the inclusion of rural study participants as well as lower ambient pollution levels in Canada. The ability to incorporate rural areas in the exposure assessment added to the variability in the studies exposure estimates, particularly for NO_2 _and O_3_, as the majority of historical NAPS measurements in Canada represent pollutant concentration in large urban areas.

The results of the retrospective air pollution modeling approach conducted here are comparable to other such studies; however, the majority of retrospective air pollution exposure assessments have been conducted solely for urban areas. For example, Bellander et al. [18] used emission data, dispersion models, and geographic information systems (GIS) to assess exposure to NO_2_, NO_x _and SO_2 _ambient air pollution during 1960, 1970 and 1980 in Stockholm, Sweden. Model evaluation using historical data was not possible, but the model was found to have high correlation (r = 0.96) with aggregated 1994-1997 data from 16 monitors. In terms of national models, Hart et al. [22] developed U.S. nationwide models of annual exposure to PM_10 _and NO_2 _from 1985 to 2000. Generalized additive models were used to predict spatial surfaces from monitoring data and GIS-derived covariates (e.g. distance to road, elevation, proportion of low-intensity residential, high-intensity residential, and industrial, commercial land use). Model performance (R^2^) for PM_10 _and NO_2 _was 0.49 and 0.88 respectively. Another national retrospective study was conducted as part of the Netherlands Cohort Study on Diet and Cancer [21]. Ambient air pollution exposures were estimated using regional (IDW monitor interpolation), urban (regression modelling), and local (road proximity) components. This approach explained 84%, 44%, 59% and 56% of the variability in averaged monitor data between 1976 and 1997 for NO_2_, NO, BS and SO_2_, respectively. The density of monitors in the Netherlands and the use of aggregated monitoring data may explain the higher model performance than seen in this study.

The exposure assessment approach presented here capitalizes on study participants' lifetime residential histories and incorporates comprehensive modelling approaches to estimate exposures to ambient air pollution and to vehicle and industrial emissions. Nevertheless, there are several limitations to this approach that may lead to exposure misclassification. Due to privacy concerns, residential addresses were coded using a standard geographic reference of 6-digit postal codes. Using a set geographic reference reduced error from changing postal codes over time; however, the spatial accuracy of postal codes varies substantially between urban and rural areas of Canada. Proximity analyses for exposures to vehicle and industrial emissions will therefore be more accurate in urban areas. The ambient air pollution exposure assessment relies on the accuracy of NAPS monitoring data, and historical monitor locations, especially in rural areas, may have been sited to capture local pollution problems. Unfortunately, no historical data were available to evaluate the representativeness of NAPS monitoring data. Due to sparse temporal and spatial PM_2.5 _monitor coverage, we created historical models based on TSP monitoring data and CMA indicator variables. While the model had good prediction, it was created from a limited number of monitoring stations from 1984 to 2000. Nevertheless, several studies have estimated PM_2.5 _successfully from TSP [6,27]. The accuracy of the final spatiotemporal PM_2.5_, NO_2 _and O_3 _surfaces is also determined from the initial concentration surface as well as fusion with historical NAPS monitoring data or predictions incorporating a linear time-trend and population density. Some anomalies exist in the current spatial surfaces, for example, high PM_2.5 _concentrations in mountainous regions and PM_2.5 _and NO_2 _in certain locations in the Prairies; however, few study participants lived in these locations and exposure misclassification is therefore limited. All historical monitors were used to adjust annual spatial pollution surfaces, which resulted in urban monitor ratios extrapolated to rural areas. Few rural monitors exist and it was not possible to restrict to rural monitors when adjusting the spatial pollution surfaces in rural areas. Exposure to vehicle emissions was based on proximity measures to a national 1996 road network and a clear limitation was the lack of historical road databases. Industrial emissions were based on a comprehensive database of industrial locations from 1970 to 1994; however, emission estimates were only available for major industries, which restricted the examination of specific industrial chemicals when minor industries were included.

## Conclusions

We conducted a comprehensive air pollution exposure assessment for a population based lung cancer case-control study of 8353 individuals using self-reported residential histories between 1975 and 1994. Incorporating residential histories was an important component of the exposure assessment approach, and necessitated the creation of national spatiotemporal air pollution models. Due to the lack of historical air pollution measurements, as well as differences in data availability between urban and rural areas, a number of modelling approaches were used to assign annual ambient PM_2.5_, NO_2 _and O_3 _concentrations, as well as proximity measures for vehicle and industrial emissions, to study participants' residential addresses. The exposure assessment methods developed here will allow subsequent epidemiological analyses to examine latency periods associated with lung cancer, include both urban and rural populations, and study the contributions of multiple ambient pollutants and local vehicle and industrial emissions to lung cancer risk in Canada. In addition, this exposure assessment has demonstrated the importance of including residential histories in long-term exposure assessments, as well as the need to carefully examine self-reported residential histories for recall bias.

## Abbreviations

PM_2.5_: Fine Particulate Matter; NO_2_: Nitrogen Dioxide; O_3_: Ozone; TSP: Total Suspended Particulates; PM_10_: Course Particulate Matter; NECSS: National Enhanced Cancer Surveillance System; NAPS: National Air Pollution Surveillance; IDW: Inverse distance weighting; AOD: Aerosol Optical Depth; MODIS: Moderate Resolution Imaging Spectroradiometer; MISR: Multiangle Imaging Sectroradiometer; GEOS-Chem: Chemical transport model; OMI: Ozone Monitoring Instrument; CHRONOS:: Canadian Regional and Hemispheric O_3 _and NO_x _System; U.S.: United States; CMA: Census Metropolitan Area; EQDB: Environmental Quality Database; SIC: Standard Industrial Classifications

## Competing interests

The authors declare that they have no competing interests.

## Authors' contributions

PH, PAD and MB designed and implemented the air pollution exposure assessment approach; KCJ implemented the NECSS case-control study; JB created the O_3 _spatial surface; and AVD, LL and RM created the PM_2.5 _and NO_2 _spatial surface. All authors have read and approved the final manuscript.

## Supplementary Material

Additional file 1 **Supplemental material**: Figure 1 Annual average (SD) pollutant concentrations from all valid historical NAPS monitoring stations that were operating for the entire study period. Figure 2 Census Metropolitan Areas (CMA's) in Canada with PM2.5 and TSP measurements used to create predictive models of historical PM2.5 concentrations. Figure 3 Yearly NOx on-road mobile emissions in Canada from 1980 to 2007 and extrapolated levels to 1970. Figure 4 NO2 exposure surfaces (note: 20 annual surfaces were created but only 5 are shown here) and locations of NAPS monitors with 50 km buffers. The study population residential years represents all residential locations between 1970 and 1994 summed within a 50 km grid. Figure 5 O3 exposure surfaces (note: 20 annual surfaces were created but only 5 are shown here) and locations of NAPS monitors with 50 km buffers. The study population residential years represents all residential locations between 1970 and 1994 summed within a 50 km grid. Figure 6 Scatter plots of measured versus predicted PM2.5, NO2 and O3 for IDW interpolation and linear regression models. Click here for file
