# Supplementary material for: Spatiotemporal air pollution exposure assessment for a Canadian population-based lung cancer case-control study
Source: Environ Health. 2012 Apr 4;11:22. doi: 10.1186/1476-069X-11-22 (PMC3372423; doi:10.1186/1476-069X-11-22)
Supplement: Additional file 1 — Supplemental material: Figure 1 Annual average (SD) pollutant concentrations from all valid historical NAPS monitoring stations that were operating for the entire study period. Figure 2 Census Metropolitan Areas (CMA's) in Canada with PM2.5 and TSP measurements used to create predictive models of historical PM2.5 concentrations. Figure 3 Yearly NOx on-road mobile emissions in Canada from 1980 to 2007 and extrapolated levels to 1970. Figure 4 NO2 exposure surfaces (note: 20 annual surfaces were created but only 5 are shown here) and locations of NAPS monitors with 50 km buffers. The study population residential years represents all residential locations between 1970 and 1994 summed within a 50 km grid. Figure 5 O3 exposure surfaces (note: 20 annual surfaces were created but only 5 are shown here) and locations of NAPS monitors with 50 km buffers. The study population residential years represents all residential locations between 1970 and 1994 summed within a 50 km grid. Figure 6 Scatter plots of measured versus predicted PM2.5, NO2 and O3 for IDW interpolation and linear regression models. [file 1476-069X-11-22-S1.PDF]

SUPPLEMENTAL MATERIAL

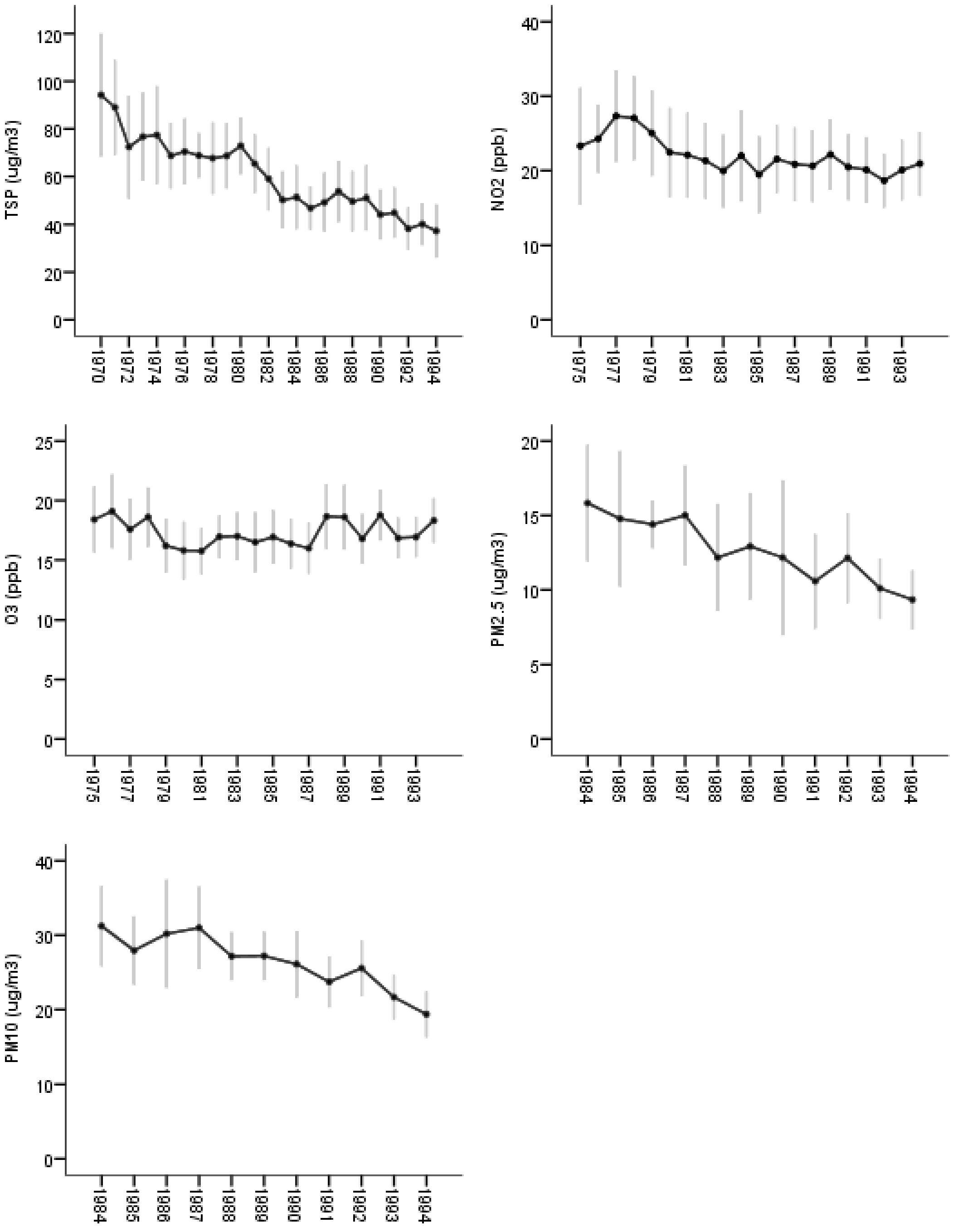

Figure 1. Annual average (SD) pollutant concentrations from all valid historical NAPS monitoring stations that were operating for the entire study period.

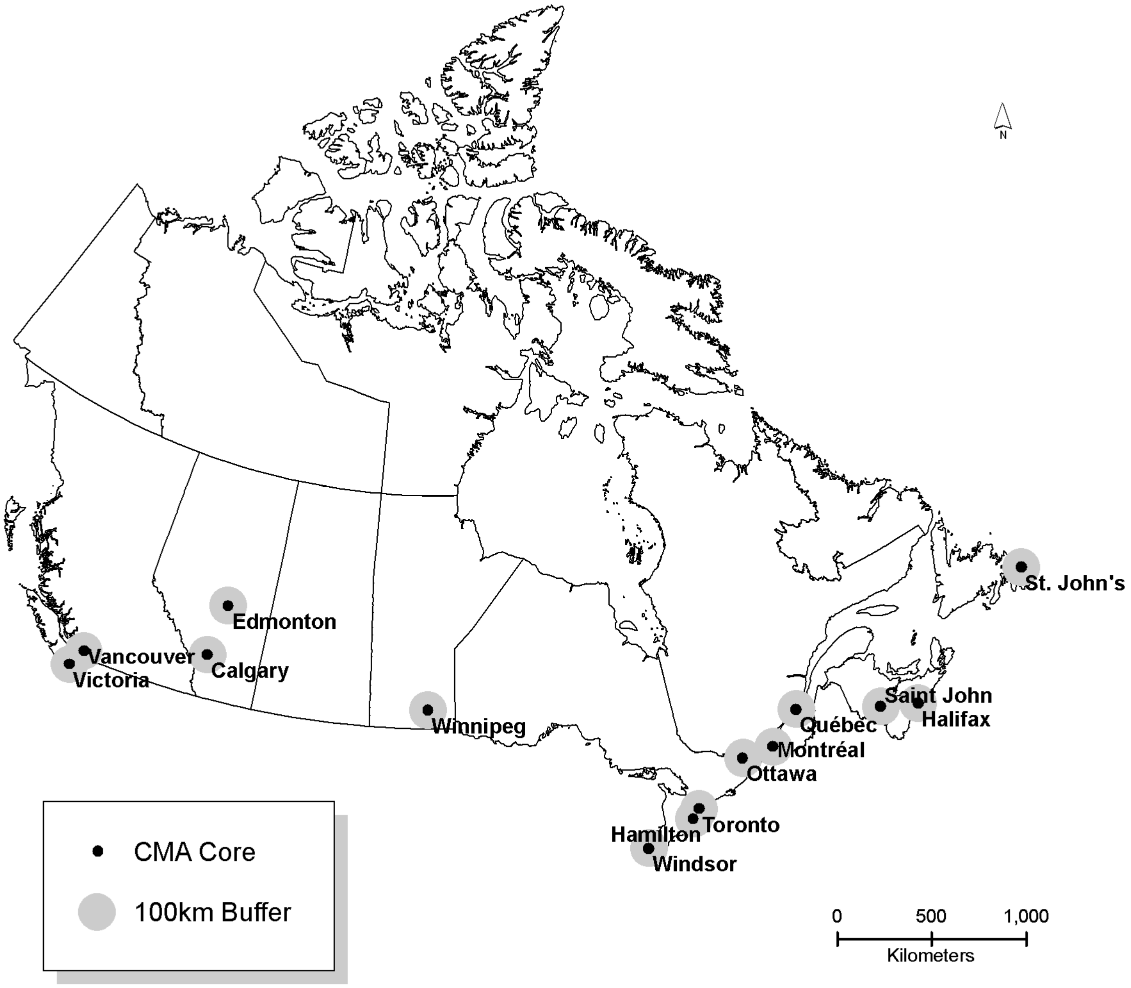

Figure 2. Census Metropolitan Areas (CMA's) in Canada with $PM_{2.5}$ and TSP measurements used to create predictive models of historical $PM_{2.5}$ concentrations.

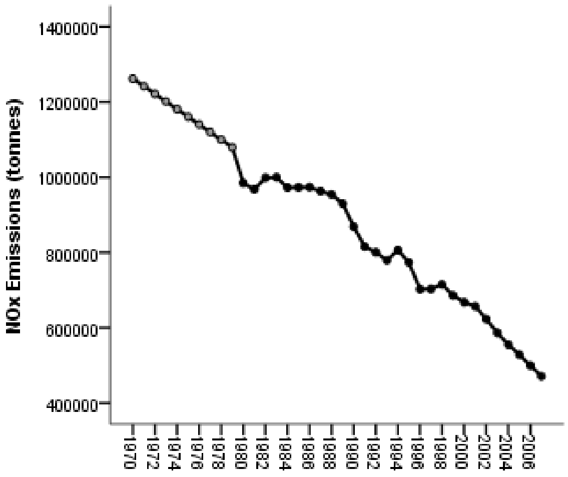

Figure 3. Yearly NO_x on-road mobile emissions in Canada from 1980 to 2007 and extrapolated levels to 1970.

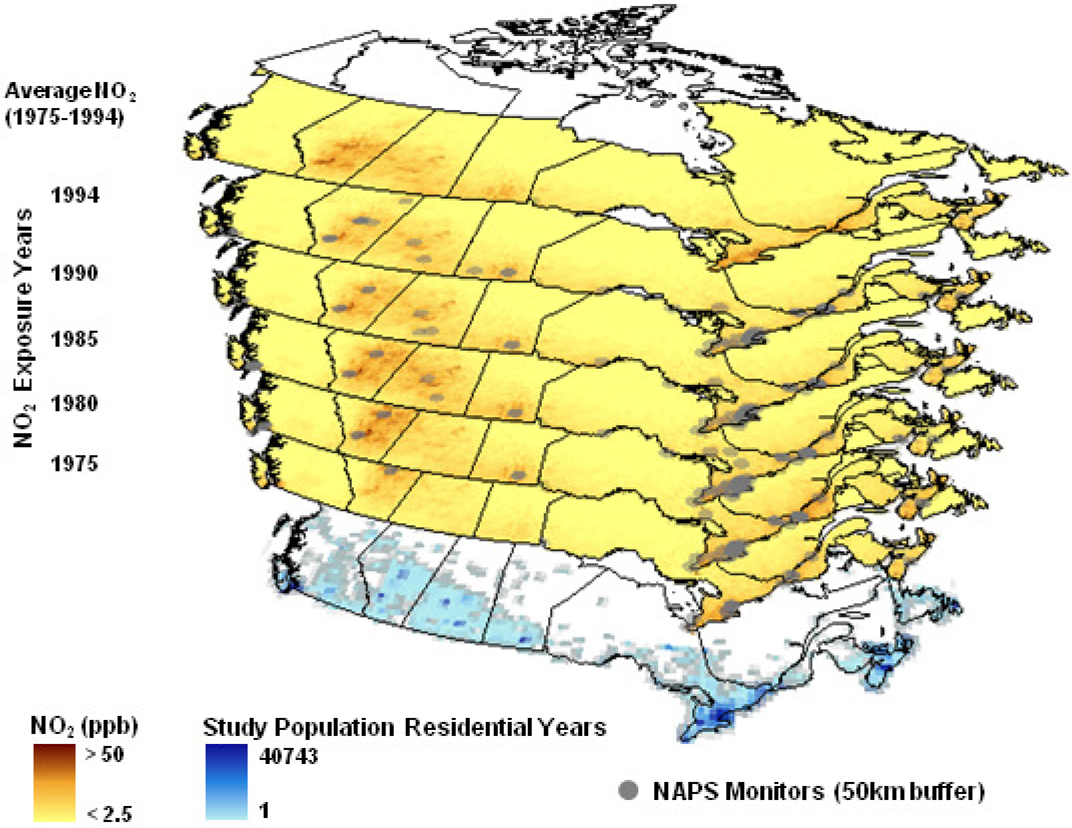

Figure 4. NO_2 exposure surfaces (note: 20 annual surfaces were created but only 5 are shown here) and locations of NAPS monitors with 50km buffers. The study population residential years represents all residential locations between 1970 and 1994 summed within a 50km grid.

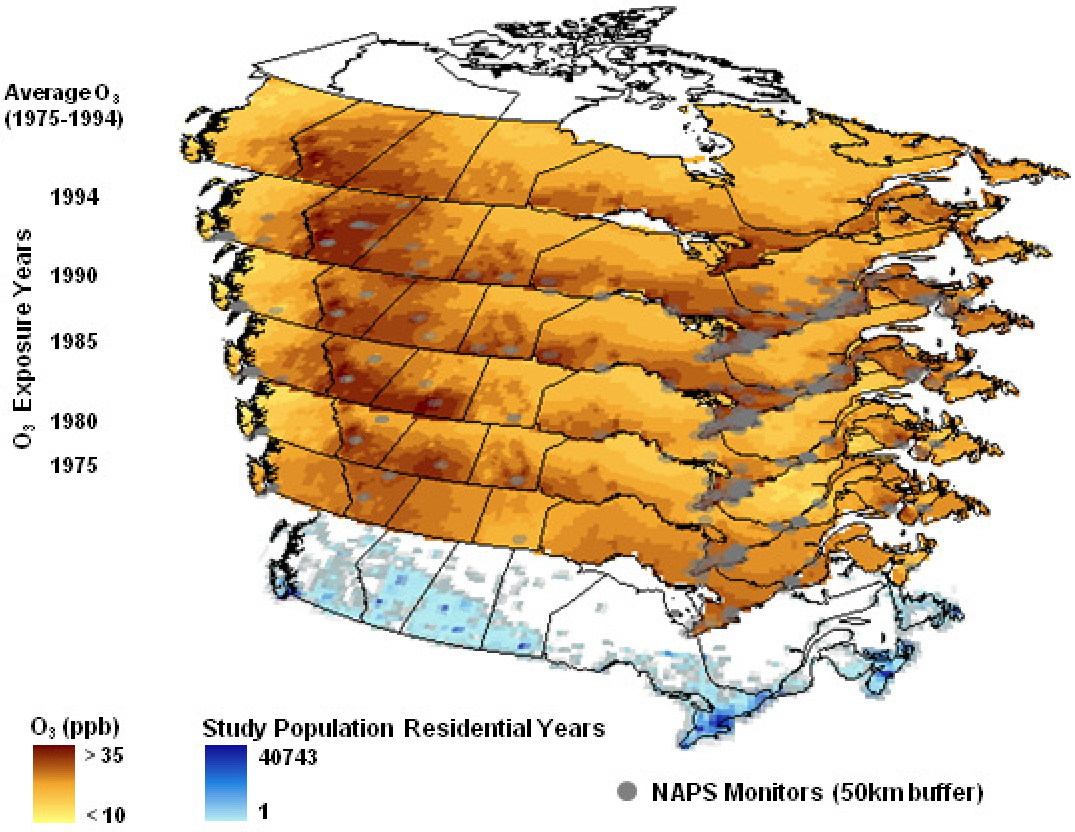

Figure 5. O_3 exposure surfaces (note: 20 annual surfaces were created but only 5 are shown here) and locations of NAPS monitors with 50km buffers. The study population residential years represents all residential locations between 1970 and 1994 summed within a 50km grid.

IDW INTERPOLATION **LINEAR MODELS**

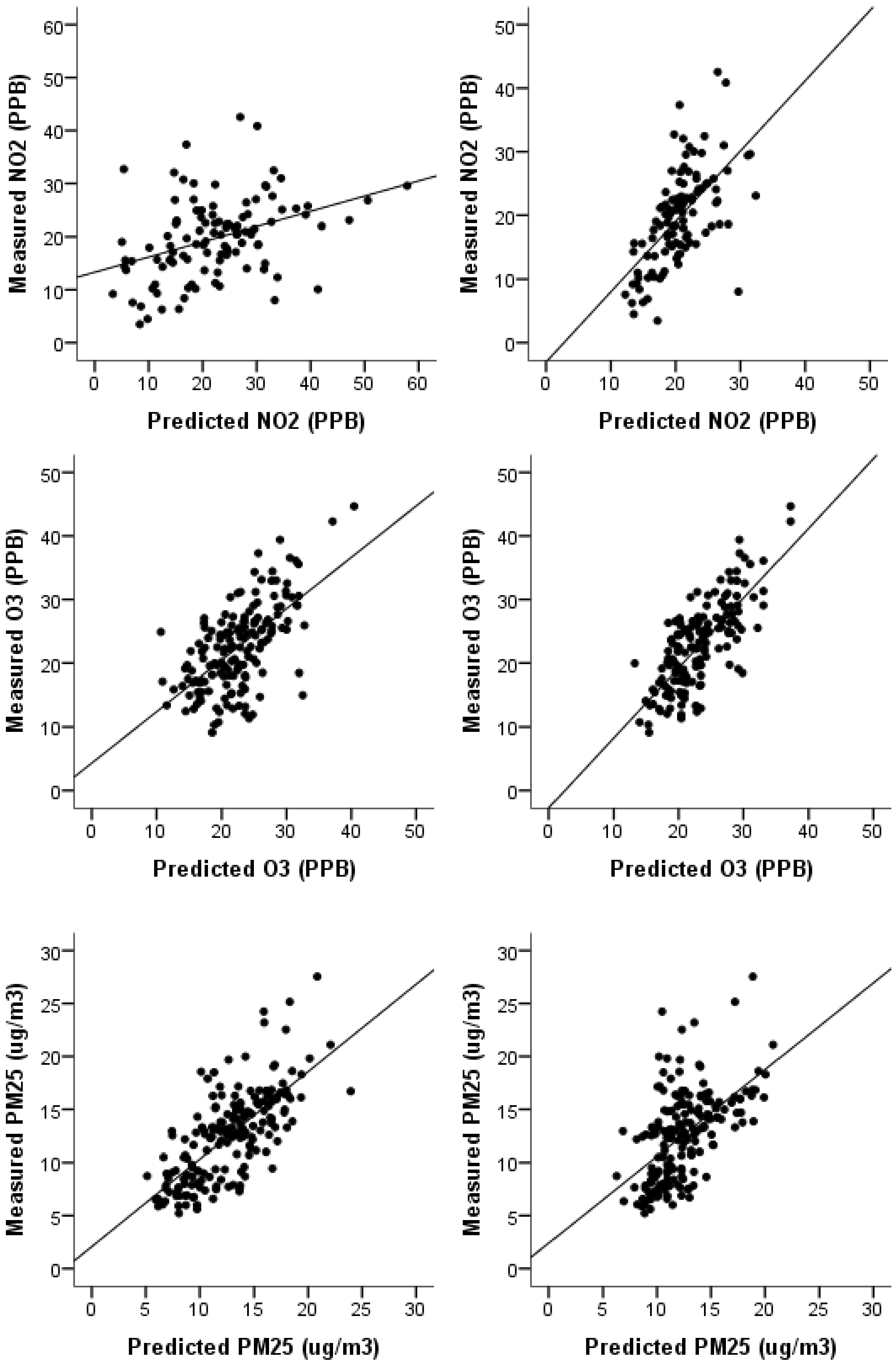

Figure 6. Scatter plots of measured versus predicted $PM_{2.5}$, NO_2 and O_3 for IDW interpolation and linear regression models.
